# Three-Layered Silk Fibroin Tubular Scaffold for the Repair and Regeneration of Small Caliber Blood Vessels: From Design to *in vivo* Pilot Tests

**DOI:** 10.3389/fbioe.2019.00356

**Published:** 2019-11-29

**Authors:** Antonio Alessandrino, Anna Chiarini, Marco Biagiotti, Ilaria Dal Prà, Giulia A. Bassani, Valentina Vincoli, Piergiorgio Settembrini, Pasquale Pierimarchi, Giuliano Freddi, Ubaldo Armato

**Affiliations:** ^1^Silk Biomaterials Srl, Lomazzo, Italy; ^2^Human Histology & Embryology Section, Department of Surgery, Dentistry, Pediatrics & Gynecology, University of Verona Medical School, Verona, Italy; ^3^Department of Vascular Surgery, San Carlo Borromeo Hospital, Milan, Italy; ^4^Institute of Translational Pharmacology, National Research Council, Rome, Italy

**Keywords:** silk fibroin, small caliber vascular graft, morphological structure, mechanical performance, *in vitro* biocompatibility, *in vivo* pilot test

## Abstract

Silk fibroin (SF) is an eligible biomaterial for the development of small caliber vascular grafts for substitution, repair, and regeneration of blood vessels. This study presents the properties of a newly designed multi-layered SF tubular scaffold for vascular grafting (SilkGraf). The wall architecture consists of two electrospun layers (inner and outer) and an intermediate textile layer. The latter was designed to confer high mechanical performance and resistance on the device, while electrospun layers allow enhancing its biomimicry properties and host's tissues integration. *In vitro* cell interaction studies performed with adult Human Coronary Artery Endothelial Cells (HCAECs), Human Aortic Smooth Muscle Cells (HASMCs), and Human Aortic Adventitial Fibroblasts (HAAFs) demonstrated that the electrospun layers favor cell adhesion, survival, and growth. Once cultured *in vitro* on the SF scaffold the three cell types showed an active metabolism (consumption of glucose and glutamine, release of lactate), and proliferation for up to 20 days. HAAF cells grown on SF showed a significantly lower synthesis of type I procollagen than on polystyrene, meaning a lower fibrotic effect of the SF substrate. The cytokine and chemokine expression patterns were investigated to evaluate the cells' proliferative and pro-inflammatory attitude. Interestingly, no significant amounts of truly pro-inflammatory cytokines were secreted by any of the three cell types which exhibited a clearly proliferative profile. Good hemocompatibility was observed by complement activation, hemolysis, and hematology assays. Finally, the results of an *in vivo* preliminary pilot trial on minipig and sheep to assess the functional behavior of implanted SF-based vascular graft identified the sheep as the more apt animal model for next medium-to-long term preclinical trials.

**Graphical Abstract:**
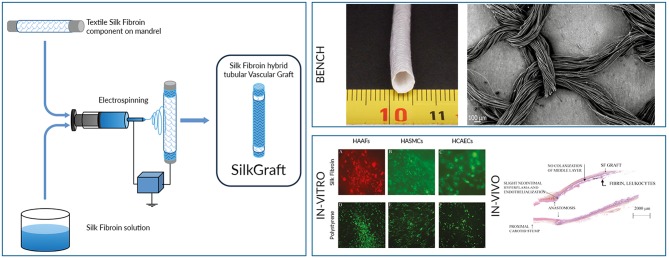
Novel hybrid textile-electrospun tubular architecture for vascular grafting, highly biocompatible, preventing fibrotic tissue responses, promising off-the-shelf solution for treating vascular diseases.

## Introduction

Cardiovascular pathologies are the leading cause of death worldwide (World Health Organization, 2012), with very high overall incidence on health expenditures. As the vascular diseases progress with age, the related burden is likely to increase with the global rise in life expectancy. Thus, the availability of grafts for the treatment of vascular diseases becomes a real and urgent need. In the vascular surgery field of either coronary or peripheral bypass procedures, there is a crucial necessity of novel viable solutions, which might complement or even replace current surgical approaches, based on autografts, or synthetic grafts (Catto et al., 2014; Hiob et al., 2017; Sugiura et al., 2017). Autografts (using native vessels such as superficial veins or rarely umbilical veins) still remain the standard clinical approach for the replacement of small diameter blood vessels. However, there are some factors which may strongly curb the use of autografts: absence of a usable graft, significative atherosclerosis of the arteries, previous usage of an autograft for surgical procedures, or angiographic approaches (Catto et al., 2014).

Nowadays, small caliber synthetic grafts are made of polyethylene terephthalate (PET) or expanded polytetrafluoroethylene (ePTFE). Their use leads to possible multiple complications like aneurysm, intimal hyperplasia, calcification, thrombosis, infection, and lack of growth potential for pediatric applications. These drawbacks are mainly correlated to the regeneration of a non-functional endothelium and a mismatch between the mechanical properties of grafts and native blood vessels leading to the development of an intimal hyperplasia with subsequent reduction of the patency rate (Catto et al., 2014 and references therein cited).

As a biodegradable and biocompatible natural polymer Silk Fibroin (SF) has the potential to become the biomaterial of choice for the development of a range of medical applications, including small caliber blood vessel grafts (Altman et al., 2003; Thurber et al., 2015; Wang et al., 2017). The starting material can be easily purified and processed in different 2D/3D shapes. It is not immunogenic in humans (preliminary proteomic data revealed that several human proteins expressed by both epithelial and connective tissue cells exhibit homology sequences with SF Armato et al., 2011) and favors angiogenesis, an essential feature for tissue repair/regeneration (Dal Prà et al., 2005).

Manufacturing technologies of SF-based small caliber tubular grafts span from filament winding (Enomoto et al., 2010; Nakazawa et al., 2011), braiding (Ding et al., 2016; Zamani et al., 2017), and knitting (Yagi et al., 2011; Yamamoto et al., 2016), which are textile techniques making use of native microfiber yarns as starting material, to electrospinning (Wang et al., 2010; Liu et al., 2011; Xiang et al., 2011), and gel spinning (Lovett et al., 2008, 2010), which lead to various formats of regenerated SF tubular scaffolds. A recent research trend is to simulate in the scaffold the three-layered structure of the native blood vessel. Thus, designing multi-layered tubular scaffolds is seen as an effective way to mimic not only the native architecture but also to approach functional features of the artery. In particular, the aim is to create regionally selective environments in favor of the infiltration, adhesion, and spreading of cells conducive to the regeneration of neo-tissues with biological features and mechanical behaviors similar to the native ones.

The simplest technical approach to create at least an additional layer is to coat the textile tubular scaffold with a film-forming biocompatible polymer (SF or gelatin) (Fukayama et al., 2015; Yamamoto et al., 2016). The use of SF microfibers as scaffold material and of SF aqueous solution as coating showed advantages in terms of enhanced *in vivo* endothelialization, which corresponded to improved graft performance (e.g., medium-to-long term patency). Addition of crosslinking agents, like poly(ethylene glycol diglycidyl ether), to aqueous SF enhanced stability, and protected the scaffold from rapid degradation when implanted *in vivo* (Yagi et al., 2011).

Building a 3D sponge-like layer on one or both sides of the tubular braided/knitted textile core may represent a step forward in approaching more complex scaffold architectures (Aytemiz et al., 2013; Liu et al., 2013, 2018; Ding et al., 2016; Zamani et al., 2017; Tanaka et al., 2018). The manufacturing techniques used by various authors, albeit with minor variations, share common steps: immersion of the tubular textile core in a mold, pouring a polymer solution, freeze-drying, and possible solvent consolidation of the just created layer. Crosslinking agents can be added to modulate porosity, strength, elasticity, and degradation rate; functional molecules, most often anti-thrombogenic agents, can also be loaded into the sponge. *In vivo* implantation up to 1 year in dogs (Aytemiz et al., 2013) and rabbits (Liu et al., 2018), and up to 3 months in rats (Tanaka et al., 2018) resulted in high patency rate, absence of thrombus, aneurysm, or infection, and a physiological level of endothelialization of the internal lumen of the graft with no signs of intimal hyperplasia.

The three-layered concept for the wall structure of the graft was approached by Enomoto et al. (2010), who fabricated a tubular scaffold by combining two kinds of native SF microfibers, i.e., a thin silk thread and cocoon filaments, which were successively arranged around a cylindrical core. The final consolidation step consisted in dipping the scaffold into a SF solution. The scaffold implanted into the abdominal aorta of male Sprague-Dawley rats showed very good patency at 1 year, organization of endothelial and medial layers, formation of *vasa-vasorum* in the adventitia, regenerating a vascular-like structure. These findings highlighted the importance of SF as a promising material to develop vascular grafts for smaller caliber blood vessels.

Wu et al. (2018) and McClure et al. (2012) engineered three-layered tubular scaffolds using only electrospinning as the manufacturing technology. Combinations of natural (SF, collagen, elastin) and biodegradable synthetic polymers [polycaprolactone, poly(L-lactide-co-caprolactone), and poly(lactide-co-glycolide)], alone or as blends, were used to build layers with finely tuned morphological and mechanical properties. The devices were fully characterized from the mechanical point of view, but the authors reported no *in vivo* functional tests. One of the devices was subcutaneously implanted for 10 weeks in rats (Wu et al., 2018) showing a propensity to promote cell infiltration from the outside environment into the interior of the graft.

Electrospinning has the ability to mimic the nanoscale properties of fibrous components (collagen and elastin fibrils) of the extracellular matrix and to realize a range of biochemical, topographical, and mechanical properties conducive to improved cell interactions (Babitha et al., 2017). Our previous studies focused on the development of small caliber vascular grafts made of electrospun SF (Marelli et al., 2009, 2010, 2012; Cattaneo et al., 2013; Catto et al., 2015). SF tubular matrices with inner diameter of about 5 mm had a pressurized burst strength of 576 ± 17 mmHg, higher than physiological and pathological pressure thresholds, but still lower than that of human arteries (~5,000 mmHg for carotids). The compliance value of about 3.5 (radial deformation/mmHg10^−2^) was considered very interesting since it is higher than synthetic grafts (<2) and closer to the physiological values for saphenous (4.4) and umbilical vein (3.7), the gold standard for autologous replacement of small caliber arteries. *In vitro* studies (Marelli et al., 2009, 2010, 2012; Catto et al., 2015) showed good integration of cells with the SF matrix, while functional implants in the abdominal aorta of Lewis rats proved short term patency of the grafts (Cattaneo et al., 2013). The cellular intimal thickening showed a structure similar to the tunica of native arterial vessel, with elastin and intimal layers reminiscent of the native inner vascular structure. Small blood vessels with the morphology of *vasa-vasorum* were found in the thin layer of tissue grown on the outer surface of the graft. Taken together, all these results showed that small caliber SF vascular grafts produced by electrospinning may be promising matrices for vascular tissue replacement, without requiring cell seeding before implantation.

This study reports the chemical, morphological, physical, and mechanical properties of a novel multi-layered SF tubular scaffold (SilkGraft). The hybrid architecture was designed to optimize not only production and pre-surgery manipulation of the device, but also stitching at the site of implantation, biological integration with host's tissues, and biomechanical performance (compliance, resistance to radial stresses, biodegradation rate, etc.). A wide range of *in vitro* cell interaction studies with human adult fibroblasts, endothelial cells, and smooth muscle cells were performed to investigate the biological response to the device. The cytokine and chemokine expression patterns were investigated to evaluate the cells' proliferative and/or pro-inflammatory attitude. The SilkGraft interaction with blood components was studied by means of the complement cascade activation assay, the change of the leucocyte and erythrocyte counts, and the hemolysis assay. Additionally, the results of *in vivo* preliminary pilot tests on large animals aimed at evaluating the handling of the device during the surgical procedure and identifying the more apt animal model for next medium-to-long term preclinical trials will be presented and discussed.

## Materials and Methods

### Fabrication of the Three-Layered Tubular Scaffold

The vascular graft is a hybrid three-layered tubular device comprising inner and outer electrospun layers (ES), and an intermediate textile layer (TEX). The TEX layer was manufactured by warp needle braiding technology using degummed SF yarn. The ES nanofibrous layers were produced via electrospinning using pupae-free silk cocoons as starting material. Cocoons were degummed in autoclave at 120°C for 30 min and extensively washed with warm water. Pure SF microfibers thus obtained were dissolved with an aqueous solution of 9.3 M lithium bromide at 60°C for 3 h. The salt was removed by dialysis and aqueous SF was cast in Petri dishes at 35°C in a ventilated oven until complete evaporation of water to produce SF films, which were then dissolved in Formic Acid (8% w_SF_/v_FA_) to prepare the spinning dope. Electrospinning was performed as previously reported (Marelli et al., 2010) using the following experimental parameters: voltage 25 kV, flow rate 0.8 ml/h, spinneret-collector distance 15 mm. Coupling of TEX and ES layers was made during electrospinning, according to a patented process (Alessandrino, 2016), with the use of an ionic liquid (1-ethyl-3-methylimidazolium acetate, Sigma Aldrich) as welding agent. Hybrid ES-TEX-ES tubular devices were finally purified by extraction with ethanol under microwave heating at 50°C for 60 min to remove processing aids, immersed in distilled water overnight, dried, packaged under laminar flow cabinet, and sterilized with ethylene oxide (EtO). Henceforth, the final three-layered SF-based ES-TEX-ES vascular graft is identified by the name “SilkGraft.”

### Materials and Scaffolds Characterization

#### Morphological and Geometrical Properties

Morphological analyses were performed with a scanning electron microscope (SEM; Zeiss EVO MA10) on Au/Pd sputter-coated samples (Desk IV, Denton Vacuum, LLC), at 10 kV acceleration voltage, 100 μA beam current, and 15 mm working distance.

Geometrical properties of SilkGraft were characterized by determining the weight per unit length, the wall thickness, and the inner diameter. Wall thickness was measured according to the ISO 7198:2016 standard method. The tubular device was cut longitudinally, flattened and measured with a thickness tester MarCator 1075R (Mahr) equipped with a constant load thickness gauge of 1 cm^2^ foot area that exerts a pressure of 1 kPa. Dry state inner diameter was determined from SEM images of tubular cross-sections mounted on stubs, using the SEM software measuring tools.

#### Amino Acid Analysis

The amino acid composition was performed after hydrolysis with HCl 6 N, under vacuum, for 24 h. Free amino acids were quantitatively determined by Ion Exchange Chromatography using external standard calibration. Samples were analyzed in duplicate.

#### Attenuated Total Reflectance Fourier Transform Infrared Spectroscopy (ATR-FTIR)

ATR-FTIR spectra were made with an ALPHA FTIR spectrometer equipped with an ATR Platinum Diamond accessory, at a resolution of 4 cm^−1^, in the infrared range of 4,000–400 cm^−1^. Spectra were corrected with a linear baseline and normalized to the CH_2_ bending peak at about 1,445 cm^−1^. This peak was selected because it's not sensitive to SF molecular conformation.

#### Differential Scanning Calorimetry (DSC)

Thermal analyses were performed with a DSC 3500 Sirius (Netzsch). Samples (3–5 mg) were closed in Aluminum pans and subjected to a heating cycle from 50 to 400°C, at a heating rate of 10°C/min, under N_2_ atmosphere (flow rate: 20 ml/min).

#### Circumferential Tensile Tests

Tests were performed according to ISO 7198:2016 using an All Electric Dynamic Test Instrument ElectroPuls E3000 (Instron), equipped with a load cell of 250 N, a thermostatic bath (BioPuls), and appropriate grips fabricated *ad hoc*. Samples (length = 10 mm, *n* = 3) were cut carefully, mounted on the grips, conditioned in water at 37°C for 5 min, and tested while submerged at a crosshead speed of 50 mm/min. Due to the difficulty in determining the correct size of the resistant cross-sectional area, the results are expressed in terms of load and not of the usual stress values.

#### Pressurized Burst Strength

The test was carried out according to ISO 7198:2016. A balloon was placed inside the tubular graft (length = 100 mm; *n* = 3) and filled with test fluid at a measured rate of pressure change until the sample burst or test was discontinued. Before testing, samples were conditioned in the test fluid (distilled water) at 37°C for 20 min. No sample pre-stretching was applied, but axial displacement (axial elongation of the sample) was allowed. The test was performed with the sample submerged in the testing fluid. A gear pump provided a flow through the sample and pressure was measured just upstream the sample. The rise in pressure and the pressure at which sample burst or test was discontinued were measured and recorded.

### *In vitro* Cellular Studies

#### Preparation of Substrates for *in vitro* Cell Cultures

SilkGraft samples were washed, transversally cut into 1.5 cm long pieces, and opened lengthwise in order to obtain small squares with an apparent surface area of about 300 mm^2^. After autoclave sterilization (121°C for 30 min), they were aseptically transferred to 2.2 cm-diameter culture plates (Falcon-Becton Dickinson). Heat-sterilized stainless-steel rings were applied onto the upper surface of the pieces to keep them flat at the bottom of the plates.

#### Pre-culture Intravital Cell Staining

Cells were stained with fluorescent lipophilic membrane dyes (tracers) such as the red-orange fluorescent DilC_18_(3) (1,1'-Dioctadecyl-3,3,3',3'-tetramethyl-indocarbocyanine perchlorate; maximum fluorescence excitation 549 nm and emission 565 nm; Thermo Fisher Scientific, USA) or the green fluorescent DiOC_18_(3) (3,3′-Dioctadecyl oxacarbocyanine perchlorate; maximum fluorescence excitation 484 nm and emission 590 nm; Thermo Fisher Scientific, USA) dissolved in DMSO according to seller's instructions. At intervals of 3 days, cultured specimens were observed under an inverted fluorescence microscope (IM 35, Zeiss) equipped with proper excitation and emission filters according to the intravital stain used, and digitally photographed with a DP10 Camera (Olympus, Japan).

#### Cell Cultures

Adult Human Coronary Artery Endothelial Cells (HCAECs), Human Aortic Smooth Muscle Cells (HASMCs), and Human Aortic Adventitial Fibroblasts (HAAFs) were provided by ScienCell Research Laboratories (Carlsbad, CA, USA). The supplier company guaranteed the cells characteristics we required via Cell Applications Inc. (San Diego, CA, USA).

For the adhesion studies, 2 × 10^4^ human intravitally pre-stained cells were separately seeded onto SilkGraft samples. HCAECs were seeded onto the inner ES layer, whereas HASMCs or HAAFs were seeded onto the outer ES layer. In parallel, the three cell types were separately seeded on 2D polystyrene plates as controls. All the tests were performed in triplicate and repeated in three separate experiments. Cell cultures were kept in an incubator at 37°C in 95 vol% air plus 5 vol% CO_2_. The growth media used were: HCAECs, ready to use Endothelial Cell Medium; HAAFs, Fibroblast Medium; HASMCs, Smooth Muscle Cell Medium (all from ScienCell Research Laboratories, USA). Every 3 days the growth media were changed with fresh ones and the cell-conditioned media collected and stored at −80°C to be subsequently analyzed. The cultures were kept going for at least 20 consecutive days.

#### Cells Counts

Cells counts on polystyrene plates were first performed using an inverted light microscope (IM35, Zeiss) (Armato et al., 1986). On the same samples, cell number was determined by means of the Cell Titer-Blue® Cell Viability Assay (Promega, USA) fluorescence assay based on the ability of living cells to convert a redox dye (resazurin) into a final fluorescent product (resorufin). Correlating microscopic findings with resorufin Promega assay data allowed to construct cell type-specific standard curves to be used to determine total cell numbers on SilkGrafts.

#### Assays of Cell Metabolites

Three metabolites (D-glucose, L-glutamine, lactate) were assayed in the cell-conditioned growth medium samples from each of the three types of cells separately cultured on SilkGraft or on polystyrene surfaces. The data corrected for actual cell numbers were expressed as means ± SE of the respective time-related cumulative curves.

Cell D-glucose consumption was assessed by means of a glucose oxidase assay using the Amplex® Red Glucose/Glucose Oxidase Assay Kit (Invitrogen-USA). L-glutamine uptake/consumption was determined using the L-glutamine assay kit developed by Megazyme (Ireland). The lactic acid release was assessed via the colorimetric enzymatic Lactate Assay Kit (Sigma Aldrich).

#### Assay of the Extracellular C-Telopeptide of Procollagen Type I

The extracellular release (and subsequent assembly of fibers) of type I collagen was assessed by evaluating the amount of the C-telopeptide, which is released into the cell-conditioned growth medium in stoichiometrically equal amounts from precursor procollagen type I molecules. The samples were assayed using the EIA kit developed by Takara Bio Inc. (Shiga, Japan). The sensitivity of this assay is 10 ng/ml.

#### Human Proinflammatory Cytokines and Chemokines Antibody Array

The secretion of various cytokines/chemokines into the growth medium was assessed by using the Human Inflammation Antibody Array, C-Series (RayBiotech-USA) in three distinct experiments. In detail, 1.0 ml medium sampled between day 18th and 20th, was used. After a treatment for 60 min with blocking solution (Odissey Blocking Buffer™ (LI-COR) with 0.05% Tween 20), the cytokines and chemokines antibody array membranes were incubated with the medium samples overnight at 4°C. Next, the membranes were washed and incubated at room temperature for 2 h with 1.0 ml of primary biotin-conjugated antibody diluted 1:250 in blocking solution. This was followed by an incubation at room temperature for 1 h with 2 ml of DyLight800-Labeled streptavidin (KPL, USA) diluted 1:7,500 in blocking solution. The immunofluorescent signals were acquired by means of an Odissey Imager™ (LI-COR) scanner and quantified using the Image Studio™ software. The resulting intensity values from each array were normalized per 1,000 cells grown on either substrate. The array kit sensitivity is 4–25 pg/ml and the coefficient of variation of the intensity of the spots is 5–10%.

### Hemocompatibility

The interaction between SilkGraft and blood components, i.e., the activation of the complement cascade and the alteration of the leucocyte count, the changes on erythrocyte count, and the presence of hemolysis, was studied according to the ISO 10993-4:2017 standard. As suggested by the regulation, the induction of thrombosis was assessed during the *in vivo* pilot trials. The tests reported below were performed in compliance with Good Laboratory Practices.

#### Activation of the Complement System

SilkGraft was incubated in human serum at 37 ± 1°C for 90 min with a surface/volume ratio of 3 cm^2^/ml. Zymosan A from *Saccharomyces cerevisiae* served as positive control, human serum alone as negative control and polypropylene material as negative reference material. Serum Complement Membrane Attack Complex (Sc5b-9) and Complement Component 3 (C3a) concentrations were determined with a commercial ELISA (MicroVue^TM^ SC5b-9 Plus EIA and MicroVue^TM^ C3a Plus EIA, Quidel Corporation).

#### Hemolysis

The blood was collected from three adult rabbits in test tube with anti-coagulant Sodium Citrate 3.2% (ratio 1+9 v/v Sodium Citrate/Blood). Equal quantities of blood from each rabbit were pooled and diluted with Mg- and Ca-free PBS to obtain a final hemoglobin concentration of 1,000 mg/dl.

For indirect contact assay, SilkGraft samples were dipped in Mg- and Ca-free PBS in order to reach a surface/volume ratio of 3 cm^2^/ml and incubated for 72 h at 50 ± 2°C. Seven milliliter of extract were added to 1 ml of diluted rabbit blood. For direct contact assay, SilkGraft samples were dipped in diluted rabbit blood and in Mg- and Ca-free PBS in order to reach a surface/volume ratio of 3 cm^2^/ml for SilkGraft/PBS and 0.14 ml/ml for diluted blood/PBS. Negative control (USP reference standard high-density polyethylene), positive control (water for injection) and blank (PBS) were tested.

All the samples (direct and indirect contact) were incubated in a water bath for 3 h at 37 ± 1°C and agitation was inverted twice every 30 min. After centrifugation at 800 G for 15 min, the concentration of hemoglobin in the supernatant was determined with the automatic chemistry analyzer Konelab 20 (DASIT).

#### Hematology

The effect of SilkGraft on red blood and white blood cells counts was evaluated after immersion in human whole blood in order to reach a surface/volume ratio of 3 cm^2^/ml and incubation for 15 min at 37 ± 1°C under dynamic conditions. An automatic counter Sysmex KX-21N (DASIT) was used. As control, the remaining part of the blood that did not come in contact with SilkGraft was used.

### *In vivo* Animal Pilot Studies

#### Animal Care

Pilot animal experiments were performed at NAMSA (Lyon, France), an AAALAC internationally accredited firm. The study protocol was approved by the NAMSA Ethical Committee and the French Ministry of Education, Higher Education, and Research. The study conditions conformed to the guidelines of the European Union's Directive EU/2010/63 for animal experiments. One sheep (Blanche du Massif Central) and one minipig (Göttingen) were used. Animals were kept under controlled conditions. The animal housing room temperature and relative humidity were recorded daily. Staff involved was properly qualified and trained. Standard veterinary medical care was also provided.

#### Pharmacological Treatment

Starting 3 days prior to the operation, the animals received daily antiaggregant treatment (sheep: oral acetylsalicylic acid, Bayer; minipig: Clopidogrel, Sanofi) to prevent thrombosis. Twenty hours before surgery the animals were weighed and Enrofloxacin (5% Baytril®, Bayer) and/or Amoxicillin (Duphamox® LA, Zoetis) were administered. After anesthesia was induced, heparin (Heparin Choay®, Sanofi) was injected into the femoral artery via an introducer sheath and Activated Clotted Time (ACT) was evaluated using a Hemochron Junior 2 (International Technidyne Corp., USA) instrument. Analgesic, anti-inflammatory drugs and antibiotic treatments were administered during surgery and the follow up period. Animals were also maintained under prophylactic anticoagulant/antiaggregant therapy for the 4 weeks of observation until termination.

#### Surgical Grafting Procedure and Follow-Up

A 2.5 cm (minipig) or 8.0 cm (sheep) segment of the carotid artery was excised and replaced by end-to-end anastomosis with a piece of sterilized SilkGraft (nominal 5 mm inner diameter) of corresponding length. Surgery was unilateral in the minipig and bilateral in the sheep. Angiography and doppler ultrasound controls were performed before and after the grafting procedure and just prior to sacrifice using Iomeron 400 (Bracco Imaging, France) as contrast medium.

After surgery, the animals were transferred to a recovery area and monitored for 1 h prior to be brought back to their housing. The supply of water and food was reinstalled. Animals were monitored for 4 weeks and then euthanized.

#### Histopathology

At autopsy, the grafts, their connected carotid stumps, and surrounding tissues were excised, endoluminal blood was removed from the carotids by gently flushing it out first with heparinized saline and then with 10% neutral buffered formalin (NBS). To complete fixation explanted samples were dipped into 10% NBS. The fixed tissues were dehydrated in ethanol solutions of increasing strength, cleared in xylene, and embedded in paraffin. Microtome sections (4–7 μm thick) were cut from each paraffin block for histopathological analysis. After removing the paraffin and rehydrating, the sections were stained with Safranin-Hematoxylin-Eosin (SHE). Histopathology analysis concerned graft endothelialization, intimal hyperplasia, thrombi, graft recellularization, potential occlusion and the presence and type of inflammatory cells.

### Statistical Analysis

Data were expressed as mean values ± SE and their level of statistical significance assessed by means of one-way ANOVA followed by Holm-Sidak's *post hoc* test. A *P* < 0.05 was taken as significant.

## Results

### Morphological, Chemical, Physical, and Mechanical Characterization

**Figure 1:**
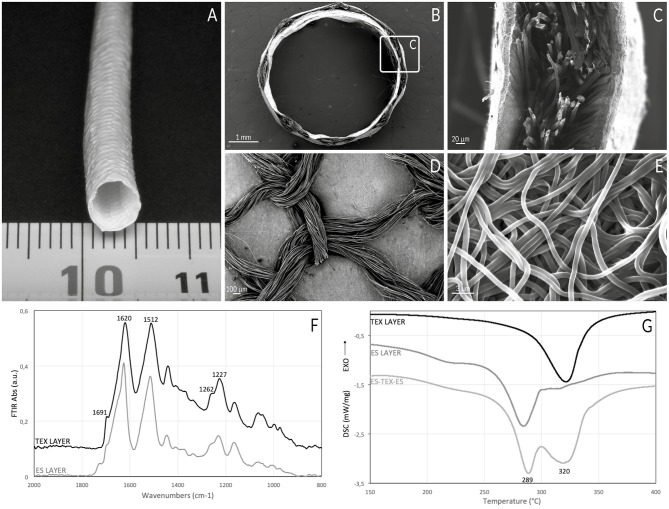
Morphological and physico-chemical structure of SilkGraft. **(A)** Picture of a SilkGraft device with 5 mm nominal inner diameter (ruler in mm). **(B)** SEM cross-section of the graft showing the two inner and outer ES layers that enclose the intermediate TEX layer (scale bar, 1 mm). **(C)** Detail of **(B)** showing a magnification of the wall structure (scale bar 20 μm). **(D)** TEX layer coupled to an ES layer, visible in the background. The texture of the braided mesh is characterized by the presence of voids (scale bar 100 μm). **(E)** SEM detail of SF nanofibers of the ES layer (scale bar, 3 μm). **(F)** ATR-FTIR spectra of TEX and ES layers in the 2,000–800 cm^−1^ range. **(G)** DSC thermograms of TEX layer, ES layer, and SilkGraft finished device (marked ES-TEX-ES) in the 150–400°C temperature range.

The SilkGraft device (Figure 1A) is a hybrid tubular structure consisting of two electrospun (ES) layers (inner and outer) and an intermediate textile (TEX) layer (Figures 1B,C). It is made of pure SF, present in the final device in form of native microfibers (TEX) with an average diameter of 12–14 μm (Figure 1D), and electrospun nanofibers (ES), whose diameter falls in the 400–600 nm range (Figure 1E) (Marelli et al., 2009).

To verify whether the processing steps, including the sterilization with EtO, altered the chemical structure and properties of the constituent biopolymer, the amino acid composition of the sterilized SilkGraft device, as well as that of two constituent materials, i.e., TEX microfibers, and ES nanofibers, was determined and compared to that of native SF microfibers. The results demonstrated that there were no differences in the amino acid composition, indicating that neither processing conditions nor sterilization with EtO (Zhao et al., 2011) altered the intrinsic properties of SF materials (Table S1).

Structural properties of SilkGraft were characterized by ATR-FTIR. The spectra of TEX microfibers and ES nanofibers (Figure 1F) display the typical profile of β-sheet crystalline materials (Marelli et al., 2010; Chiarini et al., 2016), as indicated by the position, shape, and intensity of the Amide bands (Amide I at 1,620 cm^−1^, with shoulder at 1,691 cm^−1^; Amide II at 1,512 cm^−1^; Amide III at 1,227 cm^−1^, and 1,262 cm^−1^). The crystallinity index of ES nanofibers, expressed as intensity ratio between the two Amide III components at 1,227 and 1,262 cm^−1^ (CI = I_1227_/I_1262_), was 0.58 ± 0.03, close to that determined on native SF microfibers (CI ≅ 0.60) (Chiarini et al., 2016), thus confirming the completion of the conformational transition from a prevalently random coil structure of as-spun nanofibers to a fully crystallized SF material.

Thermal properties of SilkGraft were investigated by DSC analysis. Thermograms of TEX microfibers, ES nanofibers, and final tubular device are shown in Figure 1G. TEX microfibers displayed a high thermal stability, with a main endothermic transition peaking at about 320°C, attributed to melting/degradation of highly crystalline and oriented SF microfibers (Chiarini et al., 2016). ES nanofibers, as many other regenerated SF materials, showed a marked low-temperature shift of the same transition, with a peak at about 289°C (Marelli et al., 2010). The DSC profile of the SilkGraft device was the sum of the individual components, with two discrete peaks corresponding to the thermal transitions of ES and TEX layers. The enthalpy associated with the thermal degradation of TEX (ΔH = −402 ± 41 J/g) and ES (ΔH = −307 ± 32 J/g) was used to estimate the weight contribution of each component in the final device. ES nanofibers account for about 60% by weight, while the remaining 40% is represented by the TEX layer (Table 1).

**Table 1:** Geometrical and mechanical properties of SilkGraft (nominal Ø_inner_ = 5 mm).

**Wall thickness (mm)**	**Unit weight (mg cm^**−1**^)**	**Weight (%)**		**Circumferential tensile tests**		**Burst pressure (mmHg)**
		**ES layers**	**TEX layer**	**Breaking Load (*N*)**	**Strain at break (mm/mm)**	
0.59 ± 0.03	11.9 ± 0.5	61 ± 2	39 ± 2	29.5 ± 1.0	1.60 ± 0.05	2,308 ± 88

Geometrical parameters and mechanical properties of SilkGraft with a nominal inner diameter of 5 mm are listed in Table 1. The weight per unit length of the device is very low, about 5 times lower than that of a commercial ePTFE vascular graft with similar inner diameter and wall thickness. While the ePTFE graft has a full thickness wall, the middle TEX layer of SilkGraft shows the typical structure of a braided mesh, with many voids that contribute to make the structure lighter (Figure 1D). The values of breaking load, which are about 30 times higher than those of similar grafts made only of electrospun SF nanofibers (Marelli et al., 2010), confirm that the TEX layer is the load bearing component of the graft. The contribution of the ES layers, which are solidly attached to the TEX layer through numerous welding points, is negligible. The results of burst pressure further confirm the significant contribution of the TEX layer to the mechanical performance of the multi-layered graft. The value of 2,308 mmHg is significantly larger than that of electrospun SF tubes with the same diameter (Marelli et al., 2010) and of the same order of magnitude of the values reported for internal mammary artery (3,196 ± 1,264 mmHg) and saphenous vein (1,599 ± 877 mmHg) (Konig et al., 2009).

### *In vitro* Biocompatibility Studies

The biocompatibility of SilkGraft was tested using the three most representative cell types of human peripheral arteries, namely endothelial cells (HCAECs), smooth muscle cells (HASMCs), and adventitial fibroblasts (HAAFs). To assure the full significance and relevance of the results, we chose and applied a stringent set of selective criteria. To be eligible, the human cells had to originate from disease-free human subjects, to be of recent isolation (i.e., within the second passage *in vitro*), to be free from contaminating cells of other types, to be diploid and unprocessed, to be free from viral infections (particularly HIV-1, HHVB, HHVC), and to express cell type-specific markers.

#### Cell Adhesion, Survival and Proliferation Studies

DilC_18_(3) or DiOC_18_(3) intravitally stained cells were carefully seeded on the internal (HCAECs) or external (HASMCs or HAAFs) surfaces of SilkGraft and, in parallel, onto polystyrene. Next, the fluorescent cells were observed daily under an inverted microscope for up to 3 weeks of staying *in vitro*. Examples of the three types of cells grown on SilkGraft and on polystyrene substrates are shown in Figure S1. An adhesion fraction of 61 ± 5% for each cell type was detected 3 h after seeding, which was 2.1-fold (*P* < 0.001) the fraction of the same cells adhering to polystyrene surfaces at the same time point. Morphological indicators of apoptosis (e.g., cells with a shrunk or blebbing cytoplasm and substrate-adhering or free-floating spherical apoptotic bodies) were very rarely observed. Alike data were reported previously in the case of human dermal fibroblasts isolated from adult subjects and grown on SF-covered poly(carbonate)-urethane *vs*. polystyrene (Chiarini et al., 2003). Our present findings show that with respect to a polystyrene surface, SilkGraft neatly favors the adhesion of isolated human cells. As is well-known, adhesion to a substrate favors the survival and growth of untransformed (normal) cells.

**Figure 2:**
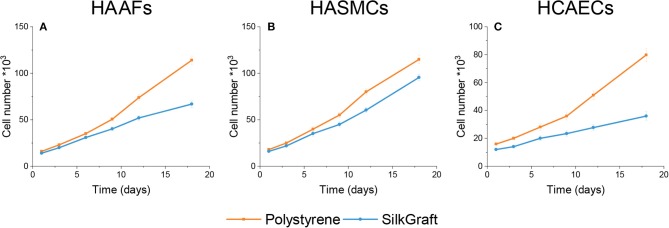
Time dependence of the absolute cell numbers of HAAF **(A)**, HASMC **(B)**, and HCAEC **(C)** cells cultured on SilkGraft and on polystyrene. Absolute cell numbers were lower on SilkGraft than on polystyrene because the available surface area was reduced due to the use of the steel ring which kept the silk substrate under water. Total cell growth differences between cells cultured on SilkGraft or on polystyrene are expressed by the areas under the corresponding curves, the statistical levels of significance of which are: for HAAFs, *P* < 0.001; for HASMCs, *P* < 0.01; and for HCAECs, *P* < 0.001.

The absolute numbers of each cell type grown on either substrate are shown and compared in Figure 2. Throughout 20 days HAAFs seeded onto SilkGraft increased their number by 4-fold, HCAECs by 3-fold, and HASMCs by 6.5-fold (*P* < 0.001 vs. 0 time in all instances). On polystyrene, the increases in numbers of the same cell types were greater, i.e., 7.5-fold for HAAFs, 5-fold for HCAECs, and nearly 7-fold for HASMCs (*P* < 0.001 vs. 0 time in all instances). However, if data are normalized to the apparent surface area available for growth (lower on SilkGraft due to the steel ring applied onto its surface), after 20 days HAAFs and HASMCs reached significantly (*P* < 0.001) greater cell densities on SilkGraft than on polystyrene, whereas HCAECs densities were similar between the two substrates (*P* > 0.05) (Table 2).

**Table 2:** Living cells density/mm^2^ of apparent surface area after 21 days of *in vitro* culture.

**Cell type**	**Polystyrene (PS) plate**	**SilkGraft (SF)**	**Ratio SF/PS**
HAAFs	363 ± 31	692 ± 63^*^	1.9 ± 0.1^*^
HASMCs	366 ± 28	1222 ± 106^*^	3.3 ± 0.2^*^
HCAECs	254 ± 23	232 ± 19^ns^	0.91 ± 0.1^ns^

Data are normalized to the apparent surface area available for growth. Levels of statistical significance of the differences between the values on SilkGraft and on polystyrene:

* *P < 0.001; ns, P > 0.05*.

#### Evaluation of Cell Metabolism

**Figure 3:**
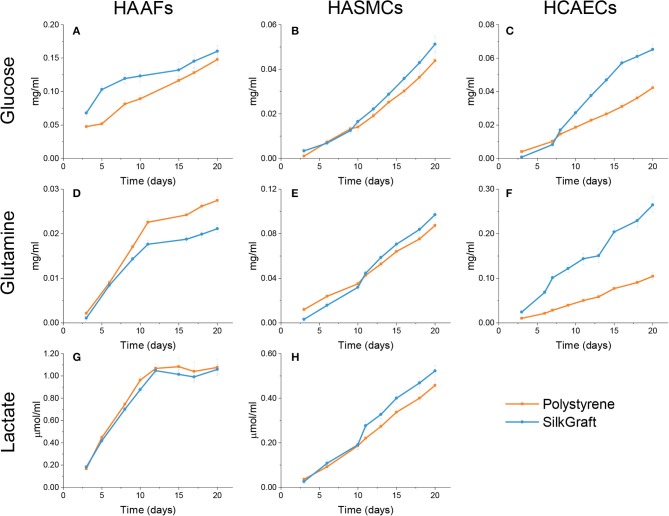
Cumulative consumption of glucose and glutamine and release of lactate. Results were normalized per 10^3^ cells. **(A–C)** The cumulative glucose consumption was higher for HAAFs (*P* < 0.05) and HCAECs (*P* < 0.001) seeded on SilkGraft, whereas it showed only marginal differences between the two substrates for HASMCs (*P* > 0.05). **(D–F)** Glutamine consumption was lower for HAAFs (*P* < 0.05) seeded on SilkGraft, similar for HASMCs (*P* > 0.05) cultured on the two substrates, and significantly larger for HCAECs (*P* < 0.001) grown on the silk substrate. **(G,H)** The cumulative amount of lactate released by HAAFs and HASMCs was the same whichever the substrate (*P* > 0.05). Lactate release could not be assessed for HCAECs because the released lactate was re-uptaken and used for metabolic purposes. The statistical analysis of these data is shown in Table 3.

The results concerning the studies on glucose and glutamine consumption, and the extracellular release of lactate were normalized per 1,000 cells and are reported in Figure 3 and Table 3. Cumulative HAAFs consumption of glucose on SilkGraft was greater than on polystyrene, the difference being much more remarkable during the first 12 days of culture *in vitro* (Figure 3A). Conversely, the cumulative HAAFs consumption of glutamine on polystyrene was greater than on SilkGraft (Figure 3D), the difference becoming remarkable only after the first 12 days. On the other hand, the release of lactate into the medium was the same whichever the substrate considered (Figure 3G).

**Table 3:** Comparison of metabolic parameters^*^ of the different cell types cultured on SilkGraft and polystyrene.

	**Polystyrene**	**SilkGraft**	**Δ%**	***P***
**HAAFs**				
Glucose uptake	1.642 ± 0.098	2.122 ± 0.150	+29.2	0.021
Glutamine uptake	0.316 ± 0.013	0.251 ± 0.012	−20.6	0.003
Lactate release	14.664 ± 0.783	13.947 ± 0.545	−4.9	0.465
Procollagen C-Telopeptide	1.500 ± 0.078	0.993 ± 0.072	−33.8	<0.001
**HCAECs**				
Glucose uptake	0.368 ± 0.026	0.572 ± 0.039	+55.4	<0.001
Glutamine uptake	0.919 ± 0.083	2.504 ± 0.198	+172.4	<0.001
**HASMCs**				
Glucose uptake	0.336 ± 0.030	0.385 ± 0.032	+14.6	0.276
Glutamine uptake	0.794 ± 0.042	0.807 ± 0.041	+1.6	0.827
Lactate release	4.016 ± 0.311	4.652 ± 0.328	+15.8	0.181

**Figure 4:**
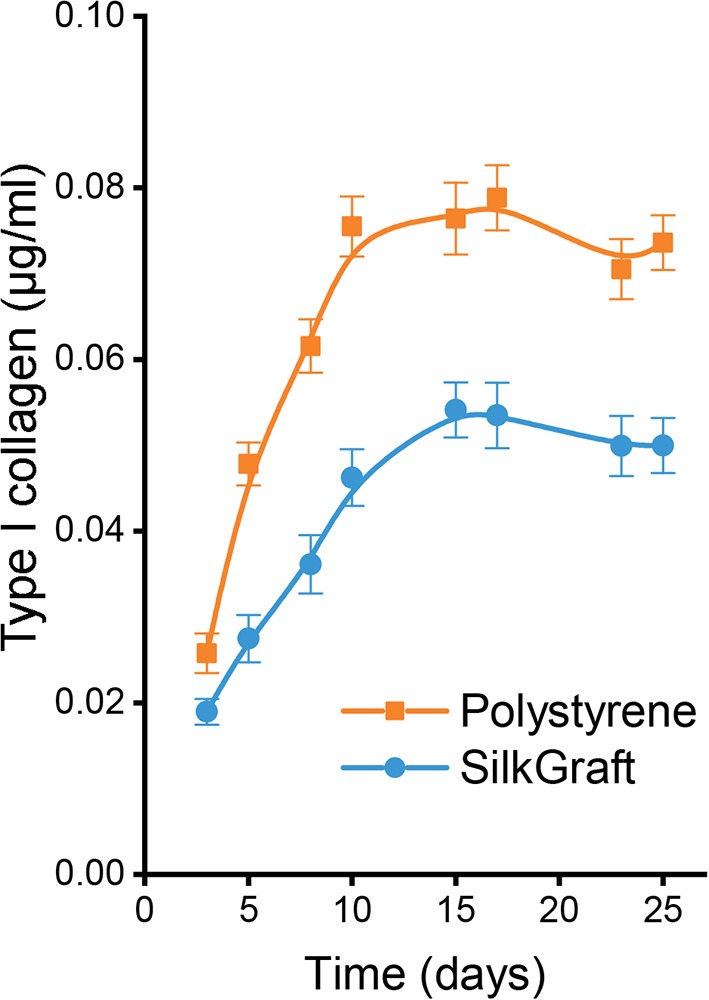
The cumulative assembly of collagen type I (indirectly measured as the stoichiometrically released C-telopeptide of procollagen) was significantly (*P* < 0.001) lower for HAAFs cultured on SilkGraft than on polystyrene surfaces. Results were normalized per 10^3^ cells. The statistical analysis of these data is shown in Table 3.

* *The parameters are the areas under the respective cumulative curves shown in Figures 3, 4*.

HASMCs cumulative consumption of glucose (Figure 3B) and glutamine (Figure 3E) and the release of lactic acid into the medium (Figure 3H) were not significantly affected by their attachment to either substrate.

Glutamine oxidation is the main source of energy for HCAECs. On SilkGraft the cumulative consumption of glutamine was typically much higher than on polystyrene (Figure 3F). The cumulative consumption of glucose was also greater than on polystyrene (Figure 3C). Undetectable levels of lactate were released into the medium by HCAECs, whichever the substrate considered, due to the known fact that endothelial cells can uptake and use lactate as an additional source of energy (Krützfeldt et al., 1990; Vegran et al., 2011).

#### Synthesis of Type I Collagen by HAAFs

Type I procollagen synthesis is a specific biomarker of HAAFs (Dal Prà et al., 2006). The release of the C-telopeptide of the procollagen is a stoichiometric index of the amount of collagen type I secreted, which subsequently undergoes fiber assembly. Thus, when HAAFs were grown on SilkGraft their cumulative release of C-telopeptide significantly decreased vs. that on polystyrene (Figure 4). A quantitative comparison of type I collagen synthesis on the two substrates is listed in Table 3.

#### Cytokine and Chemokine Expression

**Figure 5:**
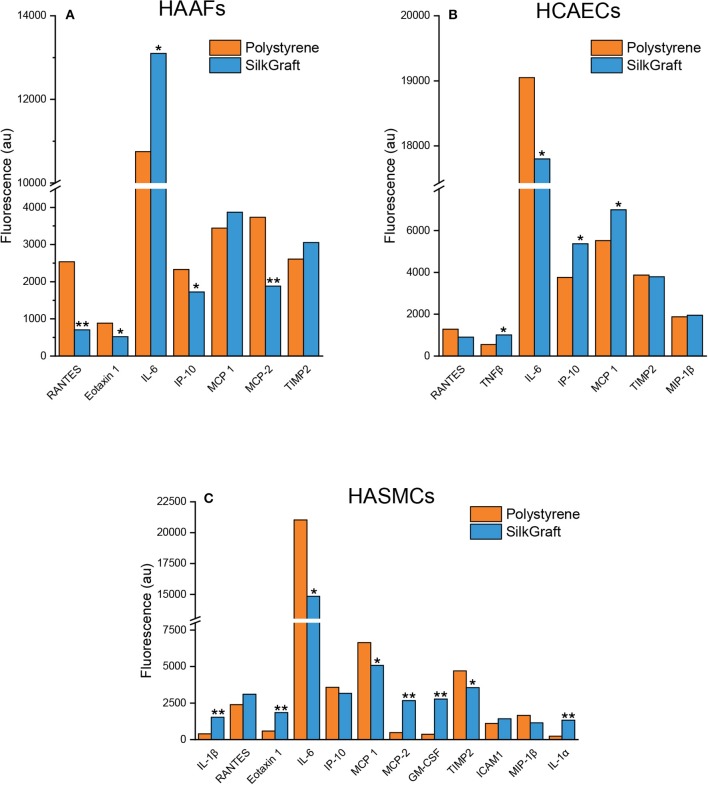
Relevant cytokines and chemokines secreted by each cell type cultured between 18 and 20 days on SilkGraft and polystyrene: HAFFs **(A)**, HCAECs **(B)**, and HASMCs **(C)**. Results of immunofluorescence intensities were normalized to 10^3^ cells. IL-6: Interleukin-6; MCP-1: Monocyte chemoattractant protein-1; TIMP-2: Tissue inhibitor of metal proteinases-2; IP-10: Interferon gamma-induced protein-10; MCP-2: Monocyte chemoattractant protein-2; Eotaxin-1; RANTES: Regulated on activation normal T cell expressed and secreted; MIP-1β: Macrophage inflammatory protein-1β; TNF-β: Tumor necrosis factor-β; GM-CSF: Granulocyte-macrophage colony stimulating factor; IL-1α: Interleukin-1α; IL-1β: Interleukin-1β; ICAM-1: Intercellular adhesion molecule-1. The bars are the mean values of three independent experiments corrected for cell numbers. *P < 0.01; **P < 0.001. SEMs, not shown, ranged between 5 and 10% of corresponding mean values.

After 20 days of staying *in vitro*, each cell type exhibited its own specific pattern of secreted cytokines and chemokines (Figure 5). No substantial qualitative differences emerged when comparing the expression patterns of each cell type in relation to the culture substrate. However, substrate-related significant differences in the quantitative secretion of some cytokines and chemokines could be detected.

HAAFs seeded on SilkGraft exhibited a higher secretion of IL-6 (+22%), MCP-1 (+13%), and TIMP-2 (+19%), whereas IP-10 (−26%), MCP-2 (−50%), Eotaxin-1 (−41%), and RANTES (−72%) were released at lesser extent than on polystyrene.

HCAECs grown on SilkGraft released higher amounts of IP-10 (+43%), MCP-1 (+27%), and TNF-β (+84%) as compared to polystyrene. RANTES and IL-6 were released to a lesser extent, while TIMP-2 and MIP-1β did not change significantly between the two substrates.

Finally, HASMCs discretely secreted a wider set of cytokines/chemokines. Interestingly, GM-CSF (+766%), MCP-2 (+557%), IL-1α (+561%), IL-1β (+390%), and Eotaxin-1 (+315%) were secreted at much higher levels on SilkGraft than on polystyrene, the secretion of ICAM-1 and RANTES only raised by +30%, whereas the release of IL-6, MIP-1β, TIMP-2, MCP-1, and IP-10 fell by −10/30% vs. the corresponding levels on polystyrene.

### Hemocompatibility

Regarding the complement activation, a statistically significant increase (*p* < 0.05) in Sc5b-9 was noted (Table S2). However, the Sc5b-9 values of SilkGraft were still inside the range of the control data of a normal standard population (334–1,672 ng/ml). Based on this, the increase was regarded as not biologically relevant. The C3a concentration was comparable to that of the negative control (Table S2). In conclusion, it can be stated that SilkGraft did not induce a biologically relevant activation of the complement system. Both indirect and direct contact hemolysis assays showed that SilkGraft exerted no hemolytic activity (hemolytic indexes <2%) (Table S3). SilkGraft did not cause any alteration of red and white blood cells counts because the difference with the respective controls was <4 and <7%, respectively (Table S4).

### Pilot Animal Studies

The purposes of the pilot studies were first to assess which animal model would be better to use for future medium-to-long term trials, and then to establish the suitability of the surgical model, to evaluate handling characteristics, aptness to stitching, patency, formation of thrombi, first signs of degradation, local tissue effects, and the general performance of SilkGraft. Resected portions of the carotid arteries of one minipig (unilateral) and one sheep (bilateral) were replaced with SilkGraft, the animals were sacrificed 4 weeks later, and histopathology of explants was examined.

Surgery was done without major difficulty on both minipig and sheep. SilkGraft preparation, handling, stitching suitability, and anastomosis capacity were overall considered as good. Moderate bleeding at the suture holes was observed. The animals survived for 4 weeks with no unwanted side effects prior to be euthanized. No graft-related clinical or macroscopic abnormalities were noted at the site of implantation or in distant organs during the test period. Ultrasound and angiographic examination at day 13 and 24 after surgery showed patent carotids, no aneurysm, dilation, dissection, blood collection or signs of infection. No stenosis was identified for the sheep, whereas a slight stenosis near the proximal anastomosis was identified for the minipig. Normal blood flow was also noted at termination. The external surface of the grafts explanted from the sheep was covered by a moderate amount of thin and soft tissue, while that recovered from the minipig was surrounded by a thicker layer of soft tissue.

**Figure 6:**
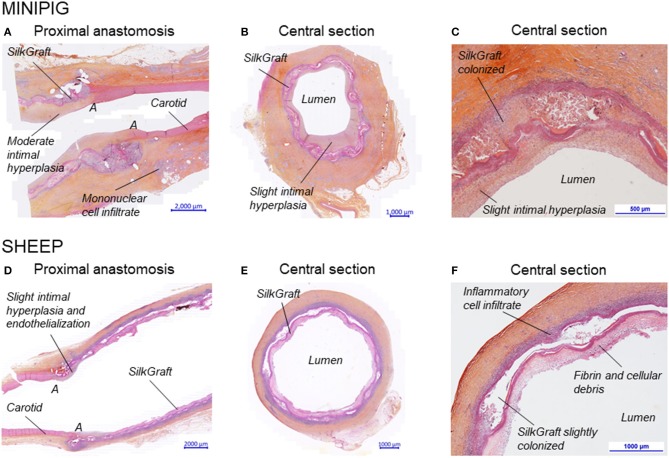
Histopathogical analysis of a SilkGraft stitched end-to-end on the carotid artery of minipig and sheep 4 weeks after grafting. Staining: Safranin-Hematoxylin-Eosin. Minipig: **(A)** Longitudinal cut in correspondence of the proximal anastomosis showing a pervious lumen (A, anastomotic line). **(B)** Representative cross-sectional cut of the central part of the graft. The lumen is pervious, though moderately reduced by an intimal hyperplasia. **(C)** Detail of the graft wall: perivascular cell infiltrates are visible; the inner and outer ES layers appear compact, whereas the TEX layer is penetrated by growing vessels and collagen-producing fibroblasts; endothelialization is visible at the inner graft surface. Sheep: **(D)** Typical longitudinal sections of the proximal end-to-end anastomotic sites (A, anastomotic line); the lumen is pervious and devoid of any stenosis. Endothelization is restricted to a 3 mm-wide area beyond the anastomosis. **(E)** Representative cross-section of the central part of the graft; the lumen is pervious while endothelization, neointimal hyperplasia, and stenosis are absent. **(F)** Detail of the graft wall: an inflammatory infiltrate envelops the graft; a newly-formed vascularized connective tissue only partly colonizes the middle TEX layer.

The histopathological analysis of the minipig explant showed that inside the graft and its attached carotid stumps the lumen was still pervious and totally devoid of thrombi (Figure 6A). Histopathology also confirmed the presence of a slight yet diffuse stenosis of the graft's lumen (Figure 6B). Endothelial-like cells lined the whole surface of the newly-formed graft's intima (Figure 6C). Collagen fibers, fibroblasts, macrophages, and multinucleated giant cells together with a lesser number of lymphocytes, plasma cells, and neutrophils filled the voids of the middle TEX layer. Colonizing cells circumferentially infiltrated the graft's outer nanofibrous ES layer. Moreover, the abundant periarterial tissue was infiltrated by lymphocytes and plasma cells. Altogether, these features suggested an ongoing foreign body response (FBR), as reported for other SF-based scaffolds (Dal Prà et al., 2005; Chiarini et al., 2016).

Grafted sheep carotids were pervious to blood flow and did not show any luminal stenosis (Figures 6D,E). Endothelization was restricted to a 3 mm-wide area beyond the end-to-end anastomotic sites. Only a thin fibrin layer and marginated leukocytes adhered to the remaining inner nanofibrous surfaces of the grafts. Also, differently from the minipig, in the long portion of the graft intervening between the anastomotic sites no newly-formed neointimal connective tissue was present. The middle TEX layer was only partially colonized by newly-formed vascularized connective tissue (Figure 6F). A thick infiltrate made up by lymphocytes, mononuclear macrophages, polynucleated giant cells, and neutrophils enveloped the graft's external nanofibrous layer, being less crowded around the proximal and distal arterial stumps. This picture indicated an ongoing FBR, but with no concurring subendothelial hyperplasia and no damage to neighboring tissues.

## Discussion

Electrospun scaffolds may suffer from some limitations when biomimicry must be coupled with a high level of mechanical performance, such as in the case of vascular grafts. An affordable solution is therefore to couple electrospinning and textile technologies (like weaving, knitting, braiding, etc.), with the aim to take advantage of both of them for realizing biomimetic scaffolds able to withstand mechanical stresses in tensile, compression, and bending modes. Zhang et al. (2017) reported the fabrication of a three-layered vascular graft where the intima and media layers were obtained by sequentially electrospinning SF and poly(L-lactide-co-ε-caprolactone), while the outer adventitia-like layer was built up by braiding SF yarns. In another study (Mi et al., 2015), a polyurethane (PU)/SF three-layered small diameter tubular scaffold was fabricated by combining electrospinning (inner PU layer) and braiding (middle SF layer) technologies. In addition, a spongy PU layer was built as third outer layer by molding and freeze drying. Both scaffolds underwent extensive mechanical and *in vitro* cytocompatibility characterization, but no *in vivo* functional tests were reported.

Coupling electrospun and textile SF matrices for the production of multi-layered tubular scaffolds is recognized as a valid approach to overcome bottlenecks due to lack of mechanical attributes of the electrospun SF scaffolds. However, the achievement of a strong adhesion between electrospun and textile layers might be a challenge, because textile surfaces onto which electrospun fibers are deposited are not homogenously flat, but present microscopic rough or sunken sites that prevent a continuous contact between the two surfaces. This results in poor adhesion strength, as demonstrated by the fact that the electrospun layer can be easily peeled off by applying a mild strength. Risks associated with poor adhesion between electrospun and textile layers may have very serious consequences on the performance of an implantable device, such as creation of morphological and mechanical discontinuities between layers, yielding and/or collapse of weaker layers, loss of geometric characteristics, thus leading to ultimate failure, especially under the stressing working conditions experienced *in vivo*.

To overcome these problems, we have developed an advanced manufacturing technology aimed at achieving an effective hybridization between electrospun and textile matrices (Alessandrino, 2016). Coating of the TEX surface with the ionic liquid before electrospinning ensures perfect welding between the electrospun and textile matrices. In fact, as soon as the first electrospun fibers reach the TEX surface, the still plastic fibers melt in contact with the ionic liquid. A dense network of strong welding points between the growing electrospun layer and the textile substrate is created. Adhesion tests showed that a force of 0.5–1 N is needed to peel off one layer from the other (Alessandrino, 2016). Therefore, the multi-layered wall of the tubular scaffold behaves as if it were made of a single piece, while harmonizing the high resistance to mechanical stresses of native SF microfibers with the enhanced biomimicking attitude of regenerated SF nanofibers. The load bearing component is the TEX layer, which confers a high mechanical resistance on the device. When stressed under wet conditions the inner and outer ES layers become very elastic. Under increasing load, they undergo extensive stretching deformation without fracturing or breaking. Interestingly, ES and TEX layers remain strongly attached to each other until the ultimate breaking point is reached, thus confirming the effectiveness of the patented coupling technology, which allows the formation of a dense network of welding points between the ES layers and the meshes of the TEX layer.

The production technology allows the fabrication of SilkGraft in a wide range of inner diameters, overlapping those of currently marketed small caliber synthetic vascular grafts. Compared to available ePTFE and PET vascular devices, SilkGraft is characterized by a design which results in a light but mechanically resistant structure. The light weight is likely to bring biological advantages because the burden of foreign material at the site of implantation is significantly reduced, thus avoiding excessive stresses to surrounding tissues during remodeling. This feature doesn't seem to negatively impact on the mechanical performance, as demonstrated by the values of circumferential breaking load and burst strength. In fact, if the breaking load reported in Table 1 is normalized to the cross-section surface, a stress value of about 3 MPa is obtained, which underestimates the real value due to the open texture of the wall of the device (see: Figure 1D). The stress value is significantly larger than that reported for the human descending mid-thoracic aorta with roughly similar inner diameter (1.72 ± 0.89 MPa) (Mohan and Melvin, 1982). Interestingly, the value of strain falls in the same range of that reported for human aorta (1.53 ± 0.28) (Mohan and Melvin, 1982).

The manufacturing protocol of SilkGraft includes various washing treatments with hydro-alcoholic solutions, which are aimed not only at achieving the complete removal of processing aids, but also at consolidating as-spun SF nanofibers that are still prevalently amorphous and more sensitive to physical, (bio)chemical, and mechanical stresses. The IR crystallinity index confirmed that nanofibers in the final device are fully crystallized. Actually, this structural feature is very important for the functionality of the device because, according to unanimous consensus, the degree of crystallinity of regenerated SF scaffolds strongly impacts on the rate of *in vivo* biodegradation (Thurber et al., 2015). In our case, the nanofibers are expected to undergo remodeling in the medium term, while in the meantime supporting neo-vascular tissue formation. To this purpose, fully crystalline ES nanofibers are more favorable to ensure a slow degradation rate in the biological environment.

The results of the *in vitro* studies on SilkGraft showed a high biocompatibility of the device, which was evaluated by seeding the three main types of cells inhabiting the arterial wall (HCAECs, HASMCs, and HAAFs). Cell adhesion to SilkGraft was in all instance superior to polystyrene, in keeping with previous observations (Chiarini et al., 2003; Dal Prà et al., 2006). The preferential energy source for both HASMCs and HCAECs was glutamine, while it was glucose for HAAFs (Wu et al., 2000; Eelen et al., 2013). HAAFs and HASMCs also secreted large amounts of lactic acid into the medium, whereas HCAECs released none. This occurred because HCAECs have the metabolic capability to convert lactate into pyruvate and to use it for oxidative metabolism, thus saving glucose which they cede to the surrounding tissues (Dal Prà et al., 2005; Chiarini et al., 2016). It should be noted that the cumulative curves of consumed glucose and glutamine and of released lactate proper of HAAFs and HASMCs exhibited similar patterns, matching the metabolic activities and the expansion of the respective cell populations.

Notably, addition of serum suppresses the contact inhibition of growth in HASMCs (Krützfeldt et al., 1990; Viñals and Pouysségur, 1999). This may explain why they reached the highest density when seeded on SilkGraft, which did not happen for HCAECs. Moreover, the observed behavioral differences could be due to unlike cell sizes (endothelial cells > fibroblasts > smooth muscle cells), morphological features, and regulatory mechanisms intrinsic to each cell type. In addition, the different characteristics of SilkGraft and polystyrene surfaces, including the adhesiveness for cells and the somewhat broader surface area offered by the nanofibers, also had an impact. Having larger sizes, the HCAECs seeded on the inner nanofibrous surface reached the confluence and the contact inhibition of growth at an earlier time and more permanently than the other two cell types. This could be beneficial because *in vivo* HCAECs, once they had reached confluence on the surface of the graft, would stop proliferating and thus keep the lumen pervious to the flowing blood. Moreover, according to our previous observations concerning HAAFs grown on SF microfibers (Chiarini et al., 2003), the present results strengthen the view that HAAFs growing on an SF substrate significantly curtail their *de novo* production of type I collagen, which translates into a remarkable antifibrogenic upshot and could be significantly relevant in the clinical settings. In other words, it can be concluded that the SF substrate in a nanofibrous format exerts a neat antifibrotic effect on HAAFs.

Some cytokines and/or chemokines are cell type-specific biomarkers. The pattern of cytokines and chemokines secreted into the growth medium under the conditions examined would reveal the proliferative and/or pro-inflammatory proclivities of the cells. In particular, released chemokines may attract circulating leukocytes. Moreover, cytokines and chemokines play important roles in several cellular processes like growth, differentiation, apoptosis, angiogenesis, inflammation, and innate immunity. Among the identified cytokines and chemokines, IL-6 stands out, being the most intensely secreted one by each of the three cell types both on SilkGraft and on polystyrene. IL-6 is considered a biomarker of cell proliferation, differentiation and survival (Morimoto et al., 1991; Krishnaswamy et al., 1998; Kyurkchiev et al., 2014). Similarly, the basal secretion of MCP-1 and/or MCP-2 is indicative of a cell proliferation capacity. MCP-1 promotes the arteriogenesis associated with the induction of Vascular Endothelial Growth Factor (VEGF)-A expression (Keeley et al., 2008). Among the other important chemokines secreted by the three cell types, we mention here IP-10, known for its antifibrotic and angiostatic properties, and RANTES protein, known for its ability to regulate leucocyte diapedesis, angiogenesis and some scarring processes (Bujak et al., 2009; Lin et al., 2018). In human aorta-derived SMCs, RANTES increased the expression of cell cycle regulatory proteins and of markers of their synthetic phenotype (Lin et al., 2018). GM-CSF is constitutively expressed by HASMCs and an elevation in GM-CSF expression associates with the HASMCs synthetic phenotype (Plenz et al., 1997). As well, the cellular adhesion molecule ICAM-1 and the HASMCs basal secretion of cytokines IL-1α and IL-1β can influence and potentiate cellular proliferation by playing an autocrine role (Braun et al., 1999; Schultz et al., 2007; Bonin et al., 2009). The low secretion of TNF-β from HCAECs on polystyrene surged significantly when the cells were grown on SilkGraft. Notably, TNF-β signals control the proper development and maintenance of endothelial cells (Zindl et al., 2009).

All the three cell types, once cultured on either SilkGraft or polystyrene, exhibited similarly low basal levels of TIMP-2, a metalloprotease blocker which regulates extracellular matrix (ECM) remodeling processes and interactions between cells and ECM, as well as cell proliferation by an autocrine mechanism (Hayakawa et al., 1994). On the other hand, it is important to note that no significant amounts of truly pro-inflammatory cytokines, such as Tumor Necrosis Factor-α (TNF-α), or of profibrotic cytokines, such as Transforming growth factor-β (TGF-β) were secreted by any of the three cell types grown on either substrate. Therefore, the patterns of cytokines and chemokines secreted by the three cell types cultured on SilkGraft suggest a proliferative attitude while neatly excluding a pro-inflammatory and/or pro-fibrogenic proclivity.

The complement system is part of the innate immune system and may be involved in promoting and accelerating hemolysis, platelet and leukocyte activation and thrombosis on device material surfaces, while hemolysis is the liberation of hemoglobin following disruption of the erythrocytes. The hemocompatibility assays demonstrated that SilkGraft did not activate the Sc5b-9 and C3a components of the complement system, did not result in hemolytic effects, and did not alter the counts of red and white blood cells. Moreover, no thrombi formation was observed during the *in vivo* pilot trials. The latter parameter will be further controlled during the on-going long-term studies (up to 1 year).

Finally, the pilot animal study showed the feasibility of using SilkGraft as small caliber vessel graft *in vivo*. Due to the size of the graft, a large animal model with vessels similar in size to human's was needed to allow appropriate placement and evaluation in view of future clinical use. Both minipig and sheep are used as appropriate animal models to evaluate performance, and local tissue effects after vascular implantation (Byrom et al., 2010). Carotid implantation was chosen to mimic clinical use and to avoid anatomical limitations (carotids are straighter and with less branches than femoral arteries). Advantages associated with using sheep as the animal model are: (i) blood vessels easily accessible and more superficial; (ii) slower endothelialization, more similar to human; (iii) ability to perform blood tests and eco-doppler with animals awake, which translates into the possibility to perform more exams with less stress for the animal; and last but not least (iv) the sheep model allows to evaluate a longer graft, up to 10 cm. Therefore, long-term studies (up to 1 year) aimed at assessing the patency and wall restructuration ability of the graft are already under course in sheep, to demonstrate the feasibility of testing SilkGraft in human clinical settings.

## Conclusions

SilkGraft is a small caliber vascular graft entirely made of pure silk fibroin. The fabrication technology was progressively refined through recursive testing and optimization procedures which led to a standardized production protocol. Particular attention was devoted to the careful selection of starting materials and processing aids (e.g., size of the SF yarn, texture and mechanical performance of the TEX layer, chemical properties and efficacy of the welding medium, etc.), as well as to fine-tuning key processing parameters (e.g., electrospinning, strategy of coupling TEX and ES layers, consolidation of the hybrid structure, final purification before sterilization, etc.). The process development led to a tubular device where the inner and outer ES layers and the middle TEX layer are perfectly integrated at the structural and functional level and respond as a single body to mechanical stresses, without showing any mutual slipping or separation. In fact, the main target was to manufacture a multi-layered scaffold characterized by an easy handling during surgery and, when implanted, able to achieve top level biomimicking performance with the surrounding living tissues while avoiding the onset of any biomechanical mismatch with the native artery.

*In vitro* studies with three main types of cells inhabiting the arterial wall, i.e., HCAECs, HASMCs, and HAAFs, showed that SilkGraft exhibited a high degree of biocompatibility and a level of cell adhesion superior to polystyrene. The trends of specific metabolic markers, like consumption of glucose and glutamine and release of acid lactic into the medium, confirmed the intense metabolic activities and the expansion of the cell populations cultured on SilkGraft coupled with a curtailed production of collagen and with no secretion of pro-inflammatory or pro-fibrotic cytokines and chemokines. Furthermore, blood hemocompatibility was corroborated by the lack of complement activation, hemolysis, and alteration of cell counts assays.

The results of pilot animal studies indicated the sheep as the model of choice to carry out the preclinical *in vivo* tests before the clinical trials in humans and confirmed that the device is easy to handle and surgically stitch. Finally, it is important to highlight that in our setting, thanks to its promising biological responses, SilkGraft is intended as an “off-the-shelf” device, no longer requiring pre-seeding with cells, thus eliminating related time delays and costs and minimizing the steps for graft preparation before implantation.

## Data Availability Statement

The raw data supporting the conclusions of this manuscript will be made available by the authors, without undue reservation, to any qualified researcher.

## Ethics Statement

The animal study was reviewed and approved by the NAMSA Ethical Committee and the French Ministry of Education, Higher Education, and Research.

## Author Contributions

This is a multi-disciplinary project that has been conducted by three groups coordinated by GF. Biomaterial development group: AA and GF led the conception and design of the project. MB, GB, and VV contributed to designing, planning, and executing all the experimental activities. Data interpretation responsibility was collectively shared by the entire group. *In vitro* preclinical study group: UA, AC, and ID were responsible for the design and planning of the *in vitro* tests. The experimental execution was performed by AC and ID, who also acquired and validated the results under the supervision of UA. *In vivo* preclinical/clinical study group: PS and PP designed the experimental approach for the *in vivo* pilot study. They shared the responsibility of selecting models, addressing surgical techniques, and evaluating the histological results. GF was responsible for drafting the text of the biomaterial development part of the work, as well as for collecting and critically revising the text contributions drafted by the other two participating groups and gathering the final approval of all authors for the publishable version. All authors ensure that questions related to the accuracy or integrity of any part of the work are appropriately investigated and resolved.

### Conflict of Interest

The study was sponsored by Silk Biomaterials srl; GF and AA are stock owners and employees of the sponsoring organization. GB, MB, and VV are employees of the sponsoring organization. UA, AC, ID, and PS are consultants of the sponsoring organization. PP was a former member of the Board of Directors of the sponsoring organization.
