# Supplementary material for: Three-Layered Silk Fibroin Tubular Scaffold for the Repair and Regeneration of Small Caliber Blood Vessels: From Design to in vivo Pilot Tests
Source: Front Bioeng Biotechnol. 2019 Nov 29;7:356. doi: 10.3389/fbioe.2019.00356 (PMC6895545; doi:10.3389/fbioe.2019.00356)
Supplement: Supplementary file 1 [file Table_1.DOCX]

# SUPPLEMENTARY MATERIALS

**Figure S1.** Major types of human adult vascular cells cultured on SilkGraft and polystyrene. (A) Human Aortic Adventitial Fibroblasts (HAAFs) intravitally pre-stained with the red-fluorescing DilC_18_(3) dye and seeded onto the external nanofibrous surface of SilkGraft pieces exhibited a typical fusiform or triangular shape. The lower fluorescence intensity of some cells is due to their more intense proliferative activity which diluteS the membrane-bound dye at each division. Original magnification: 20X. (B) Human Aortic Smooth Muscle cells (HASMCs) intravitally pre-stained with the green-fluorescing dye DiOC_18_(3) and cultured on the external nanofibrous surface of SilkGraft exhibited a prevailing thinly fusiform shape and a relative abundance of fluorescent cytoplasmic organelles. Original magnification: 20X. (C) Human Coronary Artery Endothelial cells (HCAECs) intravitally pre-stained with green-fluorescing DiOC_18_(3) and grown on the inner nanofibrous surface of SilkGraft pieces showed their typically polygonal epithelial shape as well as a wealth of organelles within the perinuclear cytoplasm. HCAECs size was larger and they were less numerous per unit surface area than HAAFs and HASMCs. Original magnification: 20X. (D-F) The three types of human cells intravitally pre-stained with DiOC_18_(3) and cultured on polystyrene surfaces after 10 days of staying *in vitro*. Original magnification: 10X. (G-I) The same cell types as seen unstained under phase-contrast microscopy; their different morphological characteristics are evident. Original magnification: 10X.

**Table S1.** Amino acid analysis of the final sterilized SilkGraft device and of its components.

| **AA**  **(mol%)** | **Native**  **SF microfibers** | **TEX layer** | **ES layer** | **SilkGraft** |
| --- | --- | --- | --- | --- |
| Asp | 1.62 ± 0.10 | 1.52 ± 0.01 | 1.51 ± 0.03 | 1.59 ± 0.04 |
| Thr | 0.95 ± 0.02 | 0.90 ± 0.09 | 0.87 ± 0.01 | 0.84 ± 0.03 |
| Ser | 10.53 ± 0.21 | 10.46 ± 0.02 | 10.42 ± 0.02 | 10.34 ± 0.02 |
| Glu | 1.35 ± 0.15 | 1.25 ± 0.03 | 1.39 ± 0.16 | 1.29 ± 0.01 |
| Pro | = | = | = | = |
| Gly | 46.49 ± 0.27 | 47.04 ± 0.32 | 46.52 ± 0.04 | 46.87 ± 0.15 |
| Ala | 29.26 ± 0.02 | 29.41 ± 0.14 | 29.40 ± 0.02 | 29.61 ± 0.14 |
| Cys | = | = | = | = |
| Val | 1.60 ± 0.20 | 1.53 ± 0.20 | 1.43 ± 0.20 | 1.58 ± 0.08 |
| Met | = | = | = | = |
| Ile | 0.62 ± 0.01 | 0.56 ± 0.07 | 0.60 ± 0.02 | 0.58 ± 0.01 |
| Leu | 1.03 ± 0.01 | 0.85 ± 0.10 | 0.97 ± 0.04 | 0.85 ± 0.11 |
| Tyr | 4.75 ± 0.16 | 4.75 ± 0.14 | 4.89 ± 0.07 | 4.69 ± 0.20 |
| Phe | 0.71 ± 0.04 | 0.69 ± 0.11 | 0.77 ± 0.05 | 0.69 ± 0.01 |
| His | 0.14 ± 0.02 | 0.13 ± 0.02 | 0.24 ± 0.01 | 0.19 ± 0.04 |
| Lys | 0.39 ± 0.07 | 0.41 ± 0.08 | 0.47 ± 0.01 | 0.39 ± 0.02 |
| Arg | 0.55 ± 0.02 | 0.49 ± 0.03 | 0.52 ± 0.06 | 0.49 ± 0.06 |

**Table S2.** Summary of the complement activation Enzyme Immunoassay

|  | **Sc5b-9** | | **C3a** | |
| --- | --- | --- | --- | --- |
| **Sample** | **(ng/ml)** | **P-values*** | **(ng/ml)** | **P-values*** |
| SilkGraft | 1582 ± 141 | 0.014 | 1158 ± 113 | 0.902 |
| Negative Control (Serum) | 629 ± 73 | / | 882 ± 144 | / |
| Negative Control Material | 843 ± 276 | / | 1151 ± 60 | / |
| Positive Control | 155*x*10^3^ ± 30*x*10^3^ | < 0.001 | 24*x*10^3^ ± 4*x*10^3^ | < 0.001 |

* P-values *vs.* Negative Control Material

**Table S3.** Summary of the Hemolysis Assays

|  | **Indirect Contact Assay** | | **Direct Contact Assay** | |
| --- | --- | --- | --- | --- |
| **Sample** | **Hemoglobin**  **(mg/dl)** | **Hemolytic Index**  **(%)*** | **Hemoglobin**  **(mg/dl)** | **Hemolytic Index**  **(%)*** |
| SilkGraft | 1.5 ± 0.1 | 1.1 | 1.0 ± 0.1 | 0.7 |
| Negative Control | 1.4 ± 0.1 | 1.0 | 1.7 ± 0.0 | 1.2 |
| Positive Control | 195.0 ± 0.3 | 140.5 | 194.6 ± 0.3 | 140.3 |
| Blank | 1.4 ± 0.1 | 1.0 | 1.3 ± 0.1 | 0.9 |

* The Hemolytic Index was calculated as follows: (hemoglobin in the supernatant)/(total hemoglobin in diluted rabbit blood with dilution factor 1:8)*100. Hemolytic Grade: *(i)* 0%-2%, non-hemolytic; *(ii)* 2%-5%, slightly hemolytic; *(iii)* > 5%, hemolytic.

**Table S4.** Summary of the Hematology Assay

|  | **SilkGraft** | **Control** | **Difference (%)** |
| --- | --- | --- | --- |
| Red Blood Cells (x10^6^/μl) | 4.65 | 4.47 | 3.87 |
| White Blood Cells (x10^3^/ml) | 6.90 | 6.65 | 3.62 |
